# Identification of Human Adenovirus in Respiratory Samples with Luminex Respiratory Virus Panel Fast V2 Assay and Real-Time Polymerase Chain Reaction

**DOI:** 10.3390/ijms17030297

**Published:** 2016-02-26

**Authors:** Susanna Esposito, Alessia Scala, Sonia Bianchini, Alberto Zampiero, Emilio Fossali, Nicola Principi

**Affiliations:** 1 Pediatric Highly Intensive Care Unit, Department of Pathophysiology and Transplantation, Università degli Studi di Milano, Fondazione IRCCS Ca’ Granda Ospedale Maggiore Policlinico, Milan 20122, Italy; a.scala19@gmail.com (A.S.); bianchini.sonia@fastwebnet.it (S.B.); alberto.zampiero@unimi.it (A.Z.); nicola.principi@unimi.it (N.P.); 2 Pediatric Emergency Unit, Fondazione IRCCS Ca’ Granda Ospedale Maggiore Policlinico, Milan 20122, Italy; emilio.fossali@policlinico.mi.it

**Keywords:** adenovirus, children, respiratory outbreak, respiratory tract infection

## Abstract

In order to compare the last version of the Respiratory Virus Panel (RVP) Fast assay for human Adenovirus (hAdv) detection with a specific real-time polymerase chain reaction (qPCR), which is considered the gold standard for hAdv detection, nasopharyngeal samples collected from 309 children (age range, four months to eight years) with respiratory tract infection were tested using the RVP Fast v2 assay (Luminex Molecular Diagnostics, Inc., Toronto, ON, Canada) and a specific TaqMan qPCR to identify hAdv DNA. The RVP Fast v2 assay detected 30/61 (49.2%) hAdv infections that had been identified by real-time qPCR, demonstrating a significantly lower detection rate (*p* < 0.001). The sensitivity of the RVP Fast v2 assay in comparison to qPCR was lower in younger children (42.9% *vs.* 57.7%; Cohen’s kappa coefficient, 0.53); in samples with co-infections (40.0% *vs.* 56.7%; Cohen’s kappa coefficient, 0.52); and in samples with hAdv type C (45.9% *vs.* 57.1%; Cohen’s kappa coefficient, 0.60). Samples with lower viral loads were associated with a significantly lower sensitivity of the RVP Fast v2 assay (35.1% *vs.* 68.2%, *p* = 0.01; Cohen’s kappa coefficients, 0.49). The RVP Fast v2 assay has important limitations for the detection of hAdv and cannot be used to evaluate whether hAdvs are the main etiologic agent responsible for an outbreak or when epidemiological studies are performed.

## 1. Introduction

Epidemics of different respiratory viruses simultaneously occur every winter season [1]. Unfortunately, the clinical symptoms are very similar regardless of the infectious agent, and only laboratory assays can clearly identify the causative virus involved in each epidemic. The Luminex Respiratory Virus Panel (RVP) Fast assay simultaneously detects 19 different viral and subtype targets in respiratory secretions and has been demonstrated since the first version (RVP classic) to be a more sensitive and less expensive method for viral detection compared to routine culture and direct fluorescent assays (DFAs) [2]. Moreover, the RVP Fast assay is rapid and straightforward to perform and can theoretically overcome some of the limitations of conventional real-time polymerase chain reaction (qPCR) assays [3]. These points underscore why the RVP Fast assay is largely used instead of qPCR for the detection of multiple viral agents in clinical practice worldwide [1,4,5]. However, direct comparisons of the RVP Fast assay with qPCR have shown that discordant results can occur for some viruses, with reduced sensitivity of the RVP Fast assay observed mainly in samples with low viral load [2,6]. This explains why the first RVP Fast Assay version was cleared by the Food and Drug Administration for the detection of only eight viruses and subtypes and for application only to nasopharyngeal swabs, leaving other specimens to be validated by the individual laboratories. The second RVP Fast Assay version v2 includes two controls to ensure an increased assay performance, but few data are available on its sensitivity and specificity in comparison to that of the qPCR assays specifically prepared for the detection of each respiratory virus [2,7,8]. Using respiratory samples, this study compared the sensitivity and specificity of the last version of the RVP Fast assay (the v2 version) for human Adenovirus (hAdv) detection with those of qPCR, which is considered the gold standard.

## 2. Results

Table 1 summarizes the comparison between the RVP Fast v2 assay and qPCR assay for hAdv detection. A total of 309 respiratory samples were evaluated, 140 of which were obtained from children <3 years of age (age range, four to 35 months) and 169 of which were collected from older subjects (age range, three to eight years). The RVP Fast v2 assay detected 30/61 (49.2%) of hAdv infections that had been identified by qPCR, demonstrating a significantly lower detection rate (*p* < 0.001). No case was identified that resulted in a positive RVP Fast v2 test and a negative qPCR test. Overall, the sensitivity and specificity of the RVP Fast v2 assay compared with the results of qPCR were 49.2% and 100%, respectively, with a positive predictive value (PPV) and negative predictive value (NPV) of 100% and 88.9%, respectively. Concordance between the two methods of hAdv identification was poor, as evidenced by the low Cohen’s kappa coefficient (0.61; 95% confidence interval [CI]: 0.49–0.73). Viral loads were <10^6^ cp/mL in 37/59 (62.7%) cases and ≥10^6^ log cp/mL in 22/59 (37.3%) cases. Among the 61 samples, 30 did not show any evidence of co-infection, whereas in the 30 additional samples, one or more viruses were detected alongside hAdv (in one sample co-infection data were not available). Rhinovirus was the most common co-infecting agent (14 samples), followed by RSV (10 samples), and influenza A (four samples). HAdv type was identified with RT-PCR in 48/61 (78.7%) cases: seven (14.6%) as type B, 37 (77.1%) as type C and four (8.3%) as other types (D, E or F).

The sensitivity of the RVP Fast v2 assay in comparison with qPCR was lower in younger children (42.9% *vs.* 57.7%), with a Cohen’s kappa coefficient of 0.53, compared with 0.70 in children ≥3 years old (*p* = 0.16 for comparison between kappa coefficients); in samples with co-infections (40.0% *vs.* 56.7%), with a Cohen’s kappa coefficient of 0.52, compared with 0.67 in samples without co-infections (*p* = 0.23 for comparison between kappa coefficients); and in samples with hAdv type C (45.9% *vs.* 57.1%), with a Cohen’s kappa coefficient of 0.60, compared with 0.72 in hAdv type B infection. Samples with lower viral loads were associated with a significantly lower sensitivity of the RVP Fast v2 assay (35.1% *vs.* 68.2%, *p* = 0.01), with a Cohen’s kappa coefficients of 0.49 for viral loads <10^6^ cp/mL compared with 0.80 for viral loads ≥10^6^ cp/mL.

## 3. Discussion

HAdv plays a significant role in pediatric respiratory tract infections, accounting for 2%–5% of overall respiratory illnesses and 4%–10% of community-acquired pneumonia [1,9]. Although most cases are mild and indistinguishable from other viral agents, acute lower respiratory tract infections (LRTIs) caused by hAdv can be severe or even fatal and are associated with a high risk of long-term respiratory sequelae [10]. Thus, early identification of hAdv-caused severe respiratory infections during outbreaks may be useful for monitoring the circulation of the virus and for planning the development of adequate preventive and therapeutic measures. This study confirms that the RVP Fast v2 assay still maintains the limitations observed when the first version of this laboratory test was evaluated [2], suggesting that there remains a high risk that large numbers of hAdv-positive respiratory samples will go unidentified if only this test is used for virus identification of respiratory infections during epidemics.

The reasons for the poor sensitivity of the hAdv RVP Fast v2 assay are not precisely defined. In this study, low viral loads were associated with a significantly lower detection rate than that observed in samples with higher viral loads. This finding suggests that for hAdv, viral load is critical for the efficient detection of positive samples using the RVP Fast v2 assay. However, this finding is in contrast to the data reported by Gadsby *et al.* [3], who did not find any association between viral load and the sensitivity of the previous version of the RVP Fast assay for hAdv detection. However, it is likely that viral load is not the only factor contributing to the sensitivity and specificity of the RVP Fast v2 assay for hAdv detection. In this study, additional samples with high viral loads that had tested positive for hAdv by qPCR were not detected by the RVP Fast v2 assay. It has been suggested that suboptimal primer binding to particular hAdv types could explain the poor sensitivity of the RVP Fast assays [4]. The primers have a mismatch with the adenovirus sequence, the amplicon has an extensive secondary structure including primer and probe annealing sites, and there are extensive interactions within and between primers. In this study, the sensitivity and specificity were found to be low for both hAdv types B and C, suggesting that all hAdv types frequently involved in causing respiratory infections could remain undetected by the RVP Fast v2 assay. However, suboptimal primer binding could still be the underlying cause of these results, as the emergence of new hAdv types and recombinant and intermediate strains occurs frequently [11], which could lead to variants that cannot be detected by the RVP Fast v2 assay. Additionally, co-infections were associated with low sensitivity, indicating that there was interference of other viruses in the detection rate of hAdv by the RVP Fast v2 assay.

## 4. Experimental Section

Nasopharyngeal samples collected from otherwise healthy children receiving treatment at the Emergency Room of the Fondazione IRCCS Ca’ Granda Ospedale Maggiore Policlinico, Milan, Italy, for respiratory tract infections in January 2014 were evaluated. The study was approved by the Ethics Committee of the Fondazione IRCCS Ca’ Granda Ospedale Maggiore Policlinico, Milan, Italy. Written informed consent of a parent or legal guardian was required, and children ≥8 years of age were asked to give their written assent. General characteristics of enrolled children were collected and archived on a previously prepared electronic chart. Nasopharyngeal secretions were collected immediately after admission to the Emergency Room using a paranasal flocked swab (one swab per child), which was stored in a tube containing 1 mL of universal transport medium (Copan, Brescia, Italy).

Viral nucleic acids were extracted from the swab using the Nuclisens EasyMAG automated extraction system (Biomeriéux, Craponne, France), and the extract was tested for respiratory viruses using the RVP Fast v2 assay (Luminex Molecular Diagnostics, Inc., Toronto, ON, Canada). This assay simultaneously detects influenza A viruses (non-specific influenza A, A/H1N1, A/H3N2, influenza A/H1N1 2009), influenza B virus, respiratory syncytial virus (RSV), parainfluenzaviruses (types 1–4), hAdv, human metapneumovirus, coronaviruses (229E, NL63, OC43 and HKU1), enterovirus/rhinovirus and human bocavirus, according to the manufacturer’s instructions (Luminex Molecular Diagnostics Inc., Toronto, ON, Canada).

Viral nucleic acid extracts were also tested using a specific HAdV plasmid by a single-plex qPCR using TaqMan Universal Master Mix II (Applied Biosystems, Foster City, CA, USA). Amplification and detection of viral DNA was performed with a 7900HT qPCR system instrument (Applied Biosystems). The real-time HAdV-specific primer sequences were as follows: 5’-GCCACGGTGGGGTTTCTAAACTT-3’, Adenoquant 1 (AQ1) and 5’-GCCCCAGTGGTCTTACATGCACATC-3’, Adenoquant 2 (AQ2). The sequence of the probe was 5’-TGCACCAGACCCGGGCTCAGGTACTCCGA-3’ (Adenoprobe) labeled with FAM on the 5’-end as a fluorescent dye and labeled with TAMRA on the 3’-end as a fluorescence quencher dye. Cycling conditions were as follows: 50 °C for 2 min, 95 °C for 8 min and 50 cycles of 95 °C for 15 s and 59 °C for 1 min. The plasmid amplified target fragment was verified by sequencing. Plasmid DNA concentrations were detected using an ND-1000 spectrophotometer (NanoDrop products, Wilmington, DE, USA).

Positive results were also quantified with a HAdV-specific qPCR, as previously described [12]. QPCR was carried out in a total reaction volume of 20 μL consisting of 10 μL of TaqMan Universal Master Mix (Applied Biosystems), 0.8 μL (0.4 mM) of each primer, 0.6 μL (0.3 mM) of the probe, 5 μL of template, and 2.8 μL of double-distilled water. The qPCR thermal cycling reaction and quantitative measurement were performed in a StepOne qPCR instrument (Applied Biosystems) using the following conditions: one cycle at 50 °C for 2 min, one cycle at 95 °C for 10 min, 45 cycles at 95 °C for 15 s, and one cycle at 60 °C for 1 min. Each run included plasmid and negative controls. Standard precautions were taken throughout the PCR process to avoid cross-contamination. Negative controls and serial dilutions of the plasmid positive control were included in every PCR assay. Finally, quantitative results were reported as DNA copies/mL of respiratory samples. The limit of detection in this work was 2.06 Log_10_. The samples were considered positive if the real-time PCR cycle threshold value was ≤42. Positive and negative controls were also included in each run.

The sensitivity, specificity, PPV, NPV, and Cohen’s kappa coefficients with 95% confidence intervals (95% CI) of the kit were calculated; a kappa coefficient higher than 0.80 was considered to be in agreement. The analyses were performed using SAS version 9.2 (Cary, NC, USA).

## 5. Conclusions

The RVP Fast v2 assay has important limitations for the detection of hAdv. This problems have limited importance in everyday patient care because no specific therapy is presently available against hAdvs. However, data collected with this study show that the RVP Fast v2 assay cannot be used to evaluate whether hAdvs are the main etiologic agent responsible for an outbreak when several respiratory viruses are simultaneously circulating or when epidemiological studies investigating the incidence of different viral respiratory infections are performed.

## Figures and Tables

**Table 1:** Comparison of the RVP Fast v2 assay and real-time PCR results for the detection of hAdv in selected subgroups.

Adenovirus	RVP Fast v2 Assay		
	Negative	Positive	Total
Subjects <3 years old			
Real-time PCR result			
Negative	105 (75.0)	0 (0.0)	105 (75.0)
Positive	20 (14.3)	15 (10.7)	35 (25.0)
Total	125 (89.3)	15 (10.7)	140 (100.0)
Sensitivity	42.9%	‒	‒
Specificity	100%	‒	‒
PPV	100%	‒	‒
NPV	84.0%	‒	‒
Cohen’s kappa (95% CI)	0.53 (0.36–0.70)	‒	‒
Subjects ≥3 years old			
Real-time PCR result			
Negative	143 (84.6)	0 (0.0)	143 (84.6)
Positive	11 (6.5)	15 (8.9)	26 (15.4)
Total	154 (91.1)	15 (8.9)	169 (100.0)
Sensitivity	57.7%	‒	‒
Specificity	100%	‒	‒
PPV	100%	‒	‒
NPV	92.9%	‒	‒
Cohen’s kappa (95% CI)	0.70 (0.53–0.86)	‒	‒
Negative or PCR viral load <10^6^ cp/mL *			
Real-time PCR result			
Negative	248 (87.0)	0 (0.0)	248 (87.0)
Positive	24 (8.4)	13 (4.6)	37 (13.0)
Total	272 (95.4)	13 (4.6)	285 (100.0)
Sensitivity	35.1%	‒	‒
Specificity	100%	‒	‒
PPV	100%	‒	‒
NPV	91.2%	‒	‒
Cohen’s kappa (95% CI)	0.49 (0.32–0.65)	‒	‒
Negative or PCR viral load ≥10^6^ cp/mL *			
Real-time PCR result			
Negative	248 (91.8)	0 (0.0)	248 (91.8)
Positive	7 (2.6)	15 (5.6)	22 (8.2)
Total	255 (94.4)	15 (5.6)	270 (100.0)
Sensitivity	68.2%	‒	‒
Specificity	100%	‒	‒
PPV	100%	‒	‒
NPV	97.3%	‒	‒
Cohen’s kappa (95% CI)	0.80 (0.65–0.94)	‒	‒
Positivity to B subtype **			
Real-time PCR result			
Negative	248 (97.2)	0 (0.0)	248 (97.2)
Positive	3 (1.2)	4 (1.6)	7 (2.8)
Total	251 (98.4)	4 (1.6)	255 (100.0)
Sensitivity	57.1%	‒	‒
Specificity	100%	‒	‒
PPV	100%	‒	‒
NPV	98.8%	‒	‒
Cohen’s kappa (95% CI)	0.72 (0.42–1.00)	‒	‒
Positivity to C subtype ***			
Real-time PCR result			
Negative	248 (87.0)	0 (0.0)	248 (87.0)
Positive	20 (7.0)	17 (6.0)	37 (13.0)
Total	268 (94.0)	17 (6.0)	285 (100.0)
Sensitivity	45.9%	‒	‒
Specificity	100%	‒	‒
PPV	100%	‒	‒
NPV	92.5%	‒	‒
Cohen’s kappa (95% CI)	0.60 (0.44–0.75)	‒	‒
No co-infections detected ^§^			
Real-time PCR result			
Negative	106 (77.9)	0 (0.0)	106 (77.9)
Positive	13 (9.6)	17 (12.5)	30 (22.1)
Total	119 (87.5)	17 (12.5)	136 (100.0)
Sensitivity	56.7%	‒	‒
Specificity	100%	‒	‒
PPV	100%	‒	‒
NPV	89.1%	‒	‒
Cohen’s kappa (95% CI)	0.67 (0.51–0.83)	‒	‒
Presence of co-infections ^§^			
Real-time PCR result			
Negative	135 (81.8)	0 (0.0)	135 (81.8)
Positive	18 (10.9)	12 (7.3)	30 (18.2)
Total	153 (92.7)	12 (7.3)	165 (100.0)
Sensitivity	40.0%	‒	‒
Specificity	100%	‒	‒
PPV	100%	‒	‒
NPV	88.2%	‒	‒
Cohen’s kappa (95% CI)	0.52 (0.34–0.70)	‒	‒

* Viral loads were not available for two PCR-positive subjects; ** Excluding PCR-positive subjects with subtypes other than “B”; *** Excluding PCR-positive subjects with subtypes other than “C”; ^§^ Information regarding co-infection was not available for eight subjects; HAdv: human adenovirus; PPV: positive predictive value; NPV: negative predictive value; real-time PCR: real-time polymerase chain reaction.
